# High frequency and diversity of parechovirus A in a cohort of Malawian children

**DOI:** 10.1007/s00705-018-04131-7

**Published:** 2019-01-22

**Authors:** Lieke Brouwer, Eveliina Karelehto, Alvin X. Han, Xiomara V. Thomas, Andrea H. L. Bruning, Job C. J. Calis, Michaël Boele van Hensbroek, Brenda M. Westerhuis, Darsha Amarthalingam, Sylvie M. Koekkoek, Sjoerd P. H. Rebers, Kamija S. Phiri, Katja C. Wolthers, Dasja Pajkrt

**Affiliations:** 1 Department of Medical Microbiology, Laboratory of Clinical Virology, Amsterdam UMC, University of Amsterdam, Meibergdreef 9, 1105 AZ Amsterdam, The Netherlands; 2 Bioinformatics Institute, Agency for Science, Technology and Research (A*STAR), Singapore, Singapore; 3 Laboratory of Applied Evolutionary Biology, Department of Medical Microbiology, Amsterdam UMC, University of Amsterdam, Amsterdam, The Netherlands; 4 Department of Pediatric Intensive Care, Emma Children’s Hospital, Amsterdam UMC, University of Amsterdam, Amsterdam, The Netherlands; 5 Department of Pediatric Infectious Diseases, Emma Children’s Hospital, Amsterdam UMC, University of Amsterdam, Amsterdam, The Netherlands; 6 School of Public Health and Family Medicine, College of Medicine, University of Malawi, Blantyre, Malawi; 7 Present Address: Department of Viroscience, Erasmus MC, Rotterdam, The Netherlands

## Abstract

Parechoviruses (PeVs) are highly prevalent viruses worldwide. Over the last decades, several studies have been published on PeV epidemiology in Europe, Asia and North America, while information on other continents is lacking. The aim of this study was to describe PeV circulation in a cohort of children in Malawi, Africa. A total of 749 stool samples obtained from Malawian children aged 6 to 60 months were tested for the presence of PeV by real-time PCR. We performed typing by phylogenetic and Basic Local Alignment Search Tool (BLAST) analysis. PeV was found in 57% of stool samples. Age was significantly associated with PeV positivity (*p* = 0.01). Typing by phylogenetic analysis resulted in 15 different types, while BLAST typing resulted in 14 different types and several indeterminate strains. In total, six strains showed inconsistencies in typing between the two methods. One strain, P02-4058, remained untypable by all methods, but appeared to belong to the recently reclassified PeV-A19 genotype. PeV-A1, -A2 and -A3 were the most prevalent types (26.8%, 13.8% and 9.8%, respectively). Both the prevalence and genetic diversity found in our study were remarkably high. Our data provide an important contribution to the scarce data available on PeV epidemiology in Africa.

**Electronic supplementary material:**

The online version of this article (10.1007/s00705-018-04131-7) contains supplementary material, which is available to authorized users.

## Introduction

Members of the genus *Parechovirus* (PeV) within the family *Picornaviridae* are small single-stranded RNA viruses. PeVs belonging to the species *Parechovirus A*, previously known as “*Human parechovirus*”, infect humans and can cause a variety of symptoms, including gastrointestinal and respiratory symptoms. Currently, 19 types of PeV-A have been distinguished, named PeV-A1-19. PeV-A3 in particular is a known cause of severe neurological disease such as meningitis and encephalitis, mainly in young children [1, 2]. For assigning new clinical strains to a type, several rules and methods have been proposed: typing based on the VP1 sequence with a 75% nucleotide (nt) sequence identity threshold [3], typing based the VP1 sequence with a 77% nt sequence identity threshold and an 87% amino acid (aa) sequence identity threshold [4], and typing based on the VP3/VP1 junction region sequence with a 82% nt and 92% aa sequence identity threshold [5]. Over the last decades, PeVs have been shown to be highly prevalent around the world. While in Europe and the USA, studies usually find a PeV prevalence (i.e. prevalence of viral RNA or infectious virus in clinical or surveillance samples) between 1 and 7% [6–11], PeV prevalence in Asia has been reported to be as high as 25% [12–15]. The most prevalent types in all of these geographical regions are PeV-A1 and -A3 [2, 7–10, 12–15]. Data on PeV circulation in Africa are scarce; only three studies have been conducted – in Kenya, Côte d’Ivoire and Ghana – finding very divergent prevalences (2%, 5.2% and 24% respectively) [16–18]. The aim of this study was to contribute to the little knowledge available on PeV circulation in Africa. For this, we tested samples collected in a cross-sectional study in Malawi for the presence of PeV.

## Materials and methods

### Patients and samples

A total of 749 fecal samples collected from Malawian children included in the SevAna study on severe anemia were included in this study [19]. The samples were collected between 2002 and 2004 from children included in one of three inclusion groups: children presenting with severe anemia (hemoglobin <5g/dl), hospital controls without severe anemia, and community controls without severe anemia. All of the included children were between 6 and 60 months of age. A questionnaire on demographic and clinical information (i.e. specific respiratory, gastrointestinal, central nervous system, and other symptoms) was completed for each participant, and clinical findings were reported. The fecal samples were stored at -80°C prior to analysis.

### Nucleic acid extraction and PeV detection

Nucleic acids were extracted from all samples by the method of Boom et al. [20]. An RT-PCR was performed as described previously [21] using primers PeV F31 and PeV K30 (Table 1). Samples with a Ct value ≤40 were considered to be PeV positive. Samples with a Ct value ≤30 were included for typing.

**Table 1:** Primers and probes used for RT-PCR (primers Parecho F31, Parecho K30 and probe WT-MGB), nested PCR (primers PeV F1, PeV R1, PeV F2 and PeV R2) and sequencing (primers PeV F2 and PeV R2)

Primer or probe	Sequence (5’-3’)	Polarity	Gene
Parecho F31	CTGGGGCCAAAAGCCA	Forward	5’UTR
Parecho K30	GGTACCTTCTGGGCATCCTTC	Reverse	5’UTR
PeV F1	TNMGNATGGGNTTYTTYCCNAAY	Forward	VP1
PeV R1	ARTARTCNARYTCRCAYTCYTC	Reverse	VP1
PeV F2	GAGTTGGACAATGCCATCTAYACNATNTGYG	Forward	VP1
PeV R2	GTTCCTGTTAGAGCTGTCTTRAANATRTCRTC	Reverse	VP1
WT-MGB (probe)	AAACACTAGTTGTAWGGCCC		5’UTR

### PeV typing and phylogenetic analysis

The complete VP1 region was sequenced for typing (Cremer et al., manuscript submitted). In short, a nested PCR was conducted using primers PeV R1, F1, R2 and F2 (Table 1). The PCR products were analyzed by agarose gel electrophoresis. Positive samples with a PCR fragment size of 1000-1100 base pairs (bp) were included for sequencing. The sequencing reaction was performed using a Big Dye Terminator Kit and primers PeV F2 and PeV R2 (Table 1). Sample sequences were assembled in CodonCode Aligner (version 6.0.2) and aligned with Mafft version 7 software (https://mafft.cbrc.jp/alignment/software/) using the L-INS-i method. Maximum-likelihood (ML) phylogenetic trees including all sample strains and reference strains from the GenBank database were constructed for the VP1 sequence (nt positions 2336 to 3037 of the reference genome sequence of the Harris PeV-A1 strain [accession no. L02791]) and the VP3/VP1 junction region (nt positions 2182 to 2437) using RaxML version 8.2.12 [22] with the generalized time-reversible (GTR) nucleotide substitution model, the gamma model of rate heterogeneity, and 1000 bootstrap replicates. A Neighbor-joining (NJ) tree including the same strains was constructed for the VP1 sequence alignment using Mega7 (p-distance, 1000 bootstrap replicates) [23]. In addition, the nucleotide sequences were compared to reference strains in the GenBank database using Nucleotide Basic Local Alignment Search Tool (BLASTn) (NCBI, https://blast.ncbi.nlm.nih.gov/ accessed 1st February 2018) with standard settings for megablast (i.e., word size 28; match/mismatch scores 1, -2; linear gap costs). For strains with BLASTn genotyping results that were either unclear or conflicted with results obtained by either phylogenetic method, we searched for the translated protein sequence using tBLASTn (word size, 6; substitution matrix, BLOSUM62; gap existence cost, 11; gap extension cost, 1). No sequences of the recently reclassified PeV-A19 were available in GenBank. As a result, for all methods, typing could only be performed for PeV-A1 through -A18. The newly obtained nucleotide sequences were deposited in the DDBJ/EMBL/GenBank nucleotide sequence databases with accession numbers MH339618-MH339740.

### Statistical analysis

Demographics were calculated in frequencies and percentages. For age, the median and interquartile range (IQR) (Q1-Q3) were calculated. The association between gender and PeV positivity, as well as between inclusion group and PeV positivity was determined by a chi-square test. The association between PeV positivity and age was calculated using the Mann-Whitney U test. Chi-square tests were performed for the association between PeV positivity and potential PeV symptoms (gastrointestinal, respiratory and central nervous system (CNS) symptoms and fever). All statistical analyses were performed using IBM SPSS Statistics 24.

## Results

### PeV prevalence in Malawian children

**Fig. 1:**
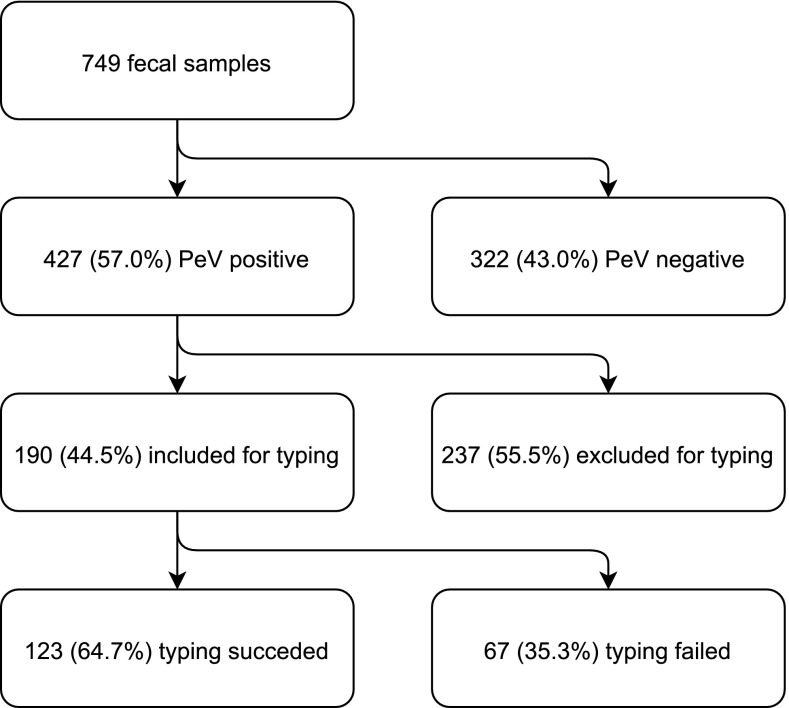
Flowchart showing all included samples

**Fig. 2:**
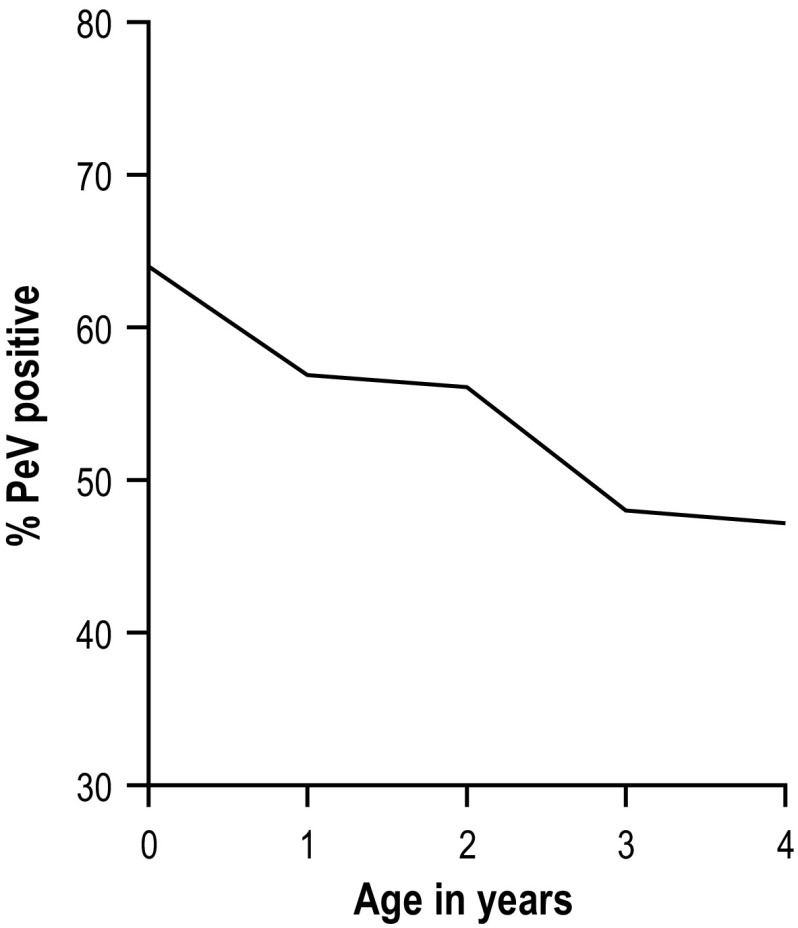
Percentage of participants (n = 740; data on age were lacking for nine of the 749 participants) with a PeV-positive stool sample by age

The baseline characteristics gender and age as well as PeV prevalence were comparable among the inclusion groups (Table 2). In total, 427 (57.0%) of the 749 samples tested positive for PeV (Table 2, Fig. 1). As data on age were lacking for nine participants, the remaining 740 participants were included for analysis on the association between age and PeV-positivity (α = 0.05). PeV positivity was significantly associated with age. PeV positive participants had a mean age of 1.8 years, and PeV-negative participants had a mean age of 2.0 years (*p* = 0.01). The proportion of PeV-positive participants was highest in children under 1 year of age (64.0%) and declined with increasing age to 47.2% in children aged 4 years (Fig. 2). PeV positivity was not associated with gender (*p* = 0.6), inclusion group (*p* = 0.5) or potential PeV-related symptoms (gastrointestinal [*p* = 0.6], respiratory [*p* = 0.9] or CNS symptoms [*p* = 0.6] or fever [*p* = 0.8]).

**Table 2:** Baseline characteristics of the 749 participants. The participants were included in the SevAna study on severe anemia between 2002 and 2004, in one of three inclusion groups: children presenting with severe anemia (hemoglobin <5 g/dl), hospital controls without severe anemia, and community controls without severe anemia. All of the children were between 6 and 60 months of age

	Severe anemia (n = 227, 30.3%)	Hospital control (n = 261, 34.8%)	Community control (n = 249, 33.2%)	Total (n = 749)*
Male gender (no., %)	107 (47.1)	133 (51.4)	120 (48.2)	371 (49.7)
Age (median, IQR)	1.30 (0.85-2.16)	1.76 (1.06-2.40)	2.00 (1.20-3.01)	1.64 (1.02 – 2.60)
PeV positive (no.,%)	128 (56.4)	142 (54.4)	148 (59.4)	427 (57.0)

*Information on inclusion group was lacking for 12 of the participants

### Genotype distribution

**Fig. 3:**
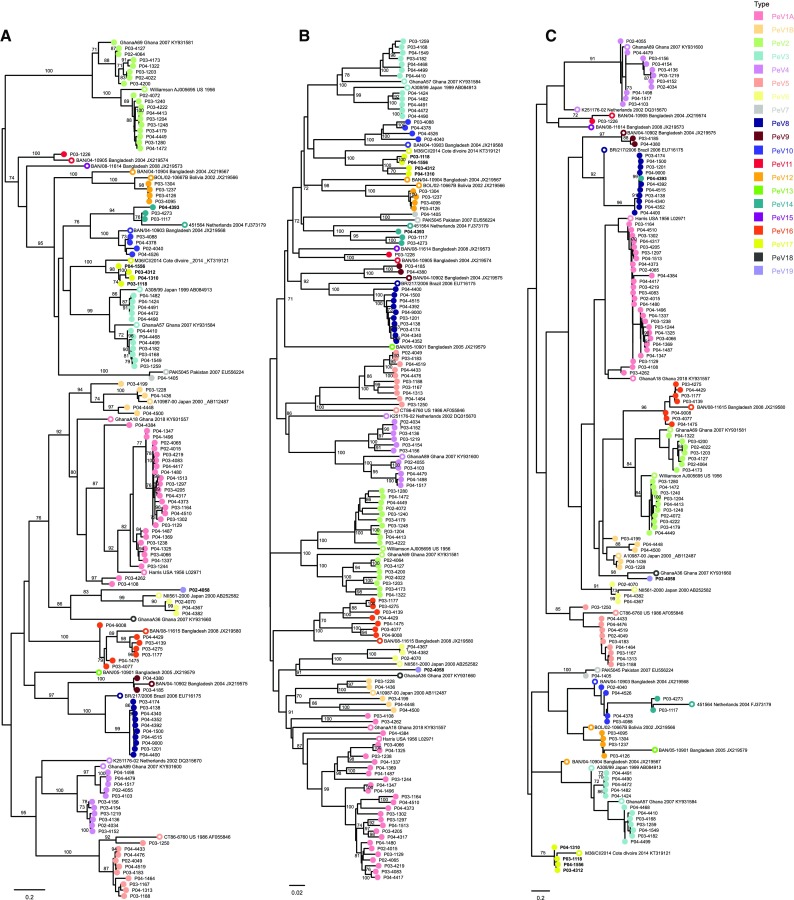
Phylogenetic trees including our study strains and selected reference strains from the GenBank database. Phylogenetic analysis was performed on the VP1 sequence alignment (A) and VP3/VP1 junction region sequence alignment (B) using the maximum-likelihood (ML) method with the generalized time-reversible (GTR) nucleotide substitution model (1000 bootstrap replicates), and on the VP1 sequence alignment using the neighbor-joining (NJ) method (p-distance, 1000 bootstrap replicates) (C). For all study strains (P0X-XXXX, circles shaded according to their predicted genotype according to phylogenetic analyses of VP1), the year of collection is indicated (P02, 2002; P03, 2003; P04, 2004). For all reference strains (open circles), the country and year of collection and the accession number are given. Strains with inconsistent typing results between the different phylogeny methods and/or BLAST are marked in bold. The untypable strain P02-4058 is labeled as PeV-A19 in this figure, according to the typing of this strain by the Picornavirus Study Group. Bootstrap values ≥70% are shown for the branches (ML-trees) or nodes (NJ-trees)

Out of 427 samples tested, 190 (44.5%) had a Ct value ≤30 and were included for additional genotyping. Good-quality sequences were obtained from 123 of 190 samples, which were typed (64.7%) (Fig. 1) using the following methods: ML phylogeny of the VP1, ML phylogeny of the VP3/VP1 junction region, NJ phylogeny of the VP1, or comparison to reference strains in GenBank using BLAST. NJ and ML phylogeny of the study strain VP1 sequences together with PeV reference strains extracted from GenBank showed that PeV-A1 was the most prevalent genotype (33/123, 26.8%), followed by PeV-A2 (17/123, 13.8%), PeV-A3 (12/123, 9.8%), and PeV-A4 (11/123, 9.0%). Other genotypes found were PeV-A5, PeV-A8 (both 10/123, 8.1%), PeV-A16 (7/123, 6.0%), PeV-A10, -A17, -A12 (4/123, 3.3%), PeV-A6, -A14 (3/123, 2.4%), PeV-A9 (2/123, 1.6%), PeV-A7 and -A11 (1/123, 0.8%) (Fig. 3, Table 3). Strain P02-4058 was distinct from all genotypes but was closest to PeV-A6, with a mean p-distance of 0.297. For PeV-A1, 28 strains grouped within the PeV-A1a cluster, while five grouped within the PeV-A1b cluster (Fig. 3).

**Table 3:** Strains typed as genotype PeV-A1 through PeV-A18 by phylogenetic analysis of the VP1 sequence (maximum-likelihood [ML] and neighbor-joining [NJ] phylogeny gave identical results) by phylogeny of the VP3/VP1 junction region and by comparing the sequences to reference strains in the GenBank database using BLAST (NCBI, https://blast.ncbi.nlm.nih.gov/, accessed February 1, 2018). Types that contained strains with inconsistent outcomes between the methods are marked in bold. The indeterminate strains include strain P02-4058, which was later typed as PeV-A19 by the Picornavirus Study Group

	Phylogeny VP1	Phylogeny VP3/VP1	BLAST
Genotype	No. of strains	No. of strains	No. of strains
PeV-A1	33	33	33
PeV-A2	17	17	17
PeV-A3	12	12	12
PeV-A4	11	11	11
PeV-A5	10	10	10
PeV-A6	3	3	3
PeV-A7	1	1	1
**PeV-A8**	10	11	10
PeV-A9	2	2	2
PeV-A10	4	4	4
PeV-A11	1	1	1
PeV-A12	4	4	4
PeV-A13	-	-	-
**PeV-A14**	3	2	3
PeV-A15	-	-	-
PeV-A16	7	7	7
**PeV-A17**	4	4	-
PeV-A18	-	-	-
**Indeterminate**	1	1	5
**Total**	123	123	123

Genotyping by ML phylogenetic analysis based on the VP1/VP3 junction region resulted in two inconsistencies compared to the analyses based on VP1: P02-4058 grouped closer to PeV-A18 (p-distance, 0.221) and -A15 (p-distance, 0.218), and P04-4393 grouped within the PeV-A8 group (Fig. 3c, Table 3). Typing the VP1 nucleotide sequences using BLAST analysis (cutoff at 77% sequence identity) resulted in inconsistencies for 11 strains compared to the phylogenetic analyses based on VP1 (P02-4058; P03-1117, -1118, -4139, -4183, -4273, -4275, -4312; P04-1310, -1475, -1556). Typing the translated aa sequences by BLAST anaysis (cutoff at 87% sequence identity) resolved the inconsistencies for six strains. The five remaining strains for which typing was not resolved included four strains that had been typed as PeV-A17 by phylogenetic analyses based on VP1 (P03-1118, P03-4312, P04-1310, P04-1556) and the untyped strain P02-4058. The PeV-A17 strains showed ≥77% nt and ≥87% aa sequence identity to both PeV-A3 and PeV-A17 strains by BLAST analysis, and therefore remained indeterminate. Strain P02-4058 showed less than 77% nt sequence identity and less than 87% aa sequence identity to any genotype and therefore remained indeterminate (Table 3 and S1).

## Discussion

We found a remarkably high PeV prevalence (57%) within a cohort of Malawian children between 2002 and 2004. This prevalence is much higher than the PeV frequencies found in Asia, Europe and North America [6–10, 12–15, 24–31]. Recently, a PeV prevalence of 24% was found in a cohort of Ghanaian children [18], pointing towards a higher PeV prevalence in Africa than in other continents. The same seems to be the case for enteroviruses, for which the reported prevalence in this and other cohorts in Africa is higher than elsewhere in the world [32–35]. We speculate that poorer hygiene than in more developed countries is a possible explanation for the high prevalence. Alternatively, other factors may have contributed to the high prevalence of PeV found in our study. Higher prevalence of PeVs was previously reported in the rainy season compared to the dry season in Ghana [18]. The fact that the vast majority of our samples were collected during the rainy season, when malaria mainly occurs, might have contributed to the high prevalence. Furthermore, PeVs are known to circulate primarily in very young populations [1], and overall, studies that included children aged up to 5 years found higher frequencies of PeV than studies that included older children and/or adults [10, 14, 17, 18, 24, 25, 30, 31]. The fact that our population consisted solely of children between 6 and 60 months of age, will therefore have contributed to the high PeV prevalence. As there was no association between PeV positivity and inclusion groups, we consider it unlikely that hospital-acquired infections or the presence of severe anemia biased the results.

Although PeV is known to cause cases of severe disease [36, 37], PeV infection is predominantly subclinical. Seroepidemiological studies have shown that the majority of children are positive for PeV neutralizing antibodies by the age of 5 years [38–41]. In line with this, we did not find a significant association between PeV infection and clinical symptoms. PeV3 in particular is known to cause severe disease, such as meningitis, encephalitis and sepsis-like illness, mainly in children under three months of age [36, 37]. However, the 11 PeV3-positive participants in our study, all above the age of six months, did not show signs of CNS infection or sepsis.

We performed typing using different, frequently used methods: NJ phylogeny based on the VP1 sequence, ML phylogeny based on the VP1 sequence, ML phylogeny based on the VP3/VP1 junction region sequence, and typing by BLAST analysis. Typing using NJ and ML phylogenetic trees identified 15 different PeV genotypes in our study; all genotypes except PeV-A13, -A15, and -A18 were found. Typing using BLAST resulted in 14 different PeV genotypes – all genotypes except PeV-A13, -A15, -A17, and -A18– while five strains had ambiguous results and were labeled indeterminate. Strain P02-4058 remained untypable by all methods. However, a similar strain had recently been submitted to the Picornavirus Study Group and was classified as PeV-A19 after minor reorganizations of the PeV-A classification (Roland Zell, personal communication, December 2018). Strain P02-4058 was therefore typed as PeV-A19. Strain P04-4393 was typed as PeV-A14 based on the VP1-sequence and as PeV-A8 based on the VP3/VP1 junction sequence. Although recombination events in the structural parts of PeV genomes are rare, they do occur and could possibly explain the different typing results obtained by different methods [42]. Our data are in line with findings in Ghana, where all genotypes were identified except PeV-A11, -A13, -A16 and -A19 [18]. We speculate that this may reflect regional differences, with a wider variety of PeV genotypes circulating in Africa than in Europe, North America and Asia, where types other than PeV-A1-6 are rarely seen [2, 7–10, 12–14]. Of the genotypes found in our study, PeV-A1, -A2 and -A3 were most prevalent. While PeV-A1 is known to circulate extensively around the world, PeV-A2 is a relatively rare genotype [9, 10, 14, 27, 29]. The high prevalence of this genotype in our study is therefore notable. While PeV-A3 is also highly prevalent worldwide [9, 10, 14, 27, 29], our results are in contrast with studies conducted in Ghana and Côte d’Ivoire, where no PeV-A3 was reported [17, 18]. Since PeV-A3 circulates more widely in children under the age of 3 months [9, 26–28], we speculate that the proportion of PeV-A3-positive samples in this study would have been even higher if children under the age of 6 months had been included.

Different rules and methods to type PeV strains have been proposed in recent years, and currently, there is no consensus regarding which specific method to use. As a result, typing is performed using different regions and lengths of the viral genome [3–5] and by using different methods, such as BLAST, NJ phylogeny and ML phylogeny [13, 14, 17, 25, 27, 30, 43]. We have shown that these methods can result in different typing results for the same viral strain, leading to inconsistent and possibly incorrect typing of PeV strains. We believe that consensus on a genotyping framework, preferably based on distinct clustering in a specialized phylogenetic analysis, is needed and would provide more accurate and consistent typing in further studies.

In conclusion, we found a high frequency of PeV circulation in a population of Malawian children. We saw multiple inconsistencies in typing of strains when comparing BLAST and phylogenetic methods. However, with all methods, we found a wide variety of genotypes, with PeV-A1, -A2 and -A3 being the most prevalent types. The presence of the higher-numbered genotypes (PeV-A7-12, -A14, -A16, -A17 and -A19) and the high prevalence of PeV-A2 are especially notable. Further studies and surveillance are needed to elucidate the impact of the high prevalence and diversity of PeV and its clinical relevance on this continent. Moreover, in the future, a consensus on a typing method may be required to avoid inconsistent typing.

## Electronic supplementary material

Below is the link to the electronic supplementary material.

Supplementary material 1 (DOCX 19 kb)

## Abbreviations

PeV: Parechovirus
(q)PCR: (Quantitative) polymerase chain reaction
Ct-value: Threshold cycle value
Bp: Base pairs
UTR: Untranslated region
VP1: Virus protein 1
nt: Nucleotide
aa: Amino acid
BLAST: Basic Local Alignment Search Tool
