# Supplementary material for: High frequency and diversity of parechovirus A in a cohort of Malawian children
Source: Arch Virol. 2019 Jan 22;164(3):799–806. doi: 10.1007/s00705-018-04131-7 (PMC6394728; doi:10.1007/s00705-018-04131-7)
Supplement: Supplementary file 1 — Supplementary material 1 (DOCX 19 kb) [file 705_2018_4131_MOESM1_ESM.docx]

|  | **Phylogeny** | | | **BLAST** | | | | | | | | | | | | | | | | | | | | | | | |
| --- | --- | --- | --- | --- | --- | --- | --- | --- | --- | --- | --- | --- | --- | --- | --- | --- | --- | --- | --- | --- | --- | --- | --- | --- | --- | --- | --- |
|  | VP1 ML | VP1 NJ | VP3/VP1 ML | ***BLASTn*** | | | | | | | | | | | | ***tBLASTn*** | | | | | | | | | | | |
|  |  |  |  | ***First match*** | | | | | | ***Second match*** | | | | | | ***First match*** | | | | | | ***Second match*** | | | | | |
|  | *Type* | *Type* | *Type* | *Type* | *Acc. Nr.* | *Similarity score (%)* | *Query cover (%)* | *E-value* | *Bit score* | *Type* | *Acc. Nr.* | *Similarity score (%)* | *Query cover (%)* | *E-value* | *Bit score* | *Type* | *Acc. Nr.* | *Similarity score (%)* | *Query cover (%)* | *E-value* | *Bit score* | *Type* | *Acc. Nr.* | *Similarity score (%)* | *Query Cover (%)* | *E-value* | *Bit score* |
| ***P03-1118*** | 17 | 17 | 17 | 3 | KJ743651 | 86 | 94 | 0.0 | 713 | 17 | KY931652 | 84 | 97 | 0.0 | 638 | 3 | KJ743672 | 100 | 100 | 2e-173 | 486 | 17 | KY931654 | 97 | 100 | 5e-169 | 475 |
| ***P03-4312*** | 17 | 17 | 17 | 3 | KJ743641 | 85 | 99 | 0.0 | 717 | 17 | KY9311652 | 82 | 97 | 2e-162 | 568 | 3 | KJ743672 | 99 | 100 | 7e-171 | 479 | 17 | KY931656 | 97 | 100 | 5e-167 | 470 |
| ***P04-1310*** | 17 | 17 | 17 | 3 | KJ743673 | 86 | 99 | 0.0 | 745 | 17 | KY931652 | 81 | 99 | 3e-156 | 547 | 3 | KJ743673 | 99 | 100 | 8e-170 | 476 | 17 | KY931656 | 97 | 99 | 3e-166 | 468 |
| ***P04-1556*** | 17 | 17 | 17 | 3 | KJ743651 | 86 | 94 | 0.0 | 712 | 17 | KY931652 | 83 | 97 | 0.0 | 630 | 3 | KJ743672 | 99 | 100 | 3e-168 | 473 | 17 | KY931656 | 96 | 100 | 8e-164 | 462 |
| ***P02-4058*** | - | - | - | 1 | KU884984 | 73 | 90 | 3e-57 | 219 | - | - | - | - | - | - | 6 | KJ743686 | 78 | 100 | 4e-136 | 391 | 18 | KY931660 | 76 | 100 | 3e-136 | 392 |
| ***P04-4393*** | 14 | 14 | *8* | *14* | *KM407608* | *79* | *88* | 8e-105 | 392 | 8 | MG026492 | 83 | 59 | 2e-95 | 361 | 14 | KY931643 | 86 | 100 | 2e-142 | 408 | 10 | JX219568 | 81 | 96 | 6e-133 | 383 |

**Supplementary Material 1:** Phylogeny results and BLAST scores for strains with inconsistent typing within and/or between phylogeny and BLAST. BLASTn and tBLASTn results (similarity scores, sequence length, e-value and bit-scores) for the first match and second match of a different type in BLAST are shown. Settings used for BLASTn were: word size 28, match/mismatch scores 1,-2, linear gap costs. Settings used for tBLASTn were: word size 6, substitution matrix BLOSUM62, gap existence cost 11, gap extension cost 1. Typing by phylogeny is given for trees of the VP1 sequence (Maximum Likelihood and Neighbor Joining) and VP3/VP1 junction sequence (Maximum likelihood. P02-4058 did not match with types other than PeV1 in nBLAST, and was untypable by phylogenetic analyses.
